# Transmission of a Protease-Secreting Bacterial Symbiont Among Pea Aphids via Host Plants

**DOI:** 10.3389/fphys.2019.00438

**Published:** 2019-04-17

**Authors:** Marisa Skaljac, Heiko Vogel, Natalie Wielsch, Sanja Mihajlovic, Andreas Vilcinskas

**Affiliations:** ^1^Branch for Bioresources, Fraunhofer Institute for Molecular Biology and Applied Ecology, Giessen, Germany; ^2^Entomology Department, Max Planck Institute for Chemical Ecology, Jena, Germany; ^3^Institute for Insect Biotechnology, Justus-Liebig University of Giessen, Giessen, Germany

**Keywords:** symbiosis, extracellular proteases, phloem sap, *Serratia symbiotica*, *Vicia faba*

## Abstract

Aphids are economically important pest insects that damage plants by phloem feeding and the transmission of plant viruses. Their ability to feed exclusively on nutritionally poor phloem sap is dependent on the obligatory symbiotic bacterium *Buchnera aphidicola*, but additional facultative symbionts may also be present, a common example of which is *Serratia symbiotica*. Many *Serratia* species secrete extracellular enzymes, so we hypothesised that *S. symbiotica* may produce proteases that help aphids to feed on plants. Molecular analysis, including fluorescence *in situ* hybridization (FISH), revealed that *S. symbiotica* colonises the gut, salivary glands and mouthparts (including the stylet) of the pea aphid *Acyrthosiphon pisum*, providing a mechanism to transfer the symbiont into host plants. *S. symbiotica* was also detected in plant tissues wounded by the penetrating stylet and was transferred to naïve aphids feeding on plants containing this symbiont. The maintenance of *S. symbiotica* by repeated transmission via plants may explain the high frequency of this symbiont in aphid populations. Proteomic analysis of the supernatant from a related but cultivable *S. symbiotica* strain cultured in liquid medium revealed the presence of known and novel proteases including metalloproteases. The corresponding transcripts encoding these *S. symbiotica* enzymes were detected in *A. pisum* and in plants carrying the symbiont, although the mRNA was much more abundant in the aphids. Our data suggest that enzymes from *S. symbiotica* may facilitate the digestion of plant proteins, thereby helping to suppress plant defense, and that the symbionts are important mediators of aphid–plant interactions.

## Introduction

Aphids are major crop pests, causing both direct feeding damage and the transmission of important plant viruses (Van Emden and Harrington, 2017). The pea aphid (*Acyrthosiphon pisum* Harris) is a model for the analysis of symbiosis, and its genome sequence was the first to be published among hemipteran insects (Consortium, 2010; Oliver et al., 2014). These species have specialised mouthparts, including a stylet that penetrates plant tissues such as sieve tubes in order to withdraw the phloem sap (Powell et al., 2006). The adaptation of aphids to this exclusive diet is facilitated by the obligatory bacterial symbiont *Buchnera aphidicola*, which compensates for the lack of nutrients by providing essential amino acids (Hansen and Moran, 2011). Aphids may also carry a variety of facultative bacterial symbionts (e.g., *Serratia symbiotica, Hamiltonella defensa*, and *Regiella insecticola*) that act as mutualists or parasites depending on the context of the environmental interactions (Oliver et al., 2010, 2014).

Facultative symbionts are found in multiple aphid tissues (including the haemolymph, gut, and reproductive system), and are sometimes co-localised with *B. aphidicola* within specialised structures known as bacteriomes (Moran et al., 2005; Skaljac et al., 2018). Most symbiotic bacteria (obligatory and facultative) are maternally inherited, whereas the extracellular and scattered localization of facultative symbionts facilitates their horizontal transfer, promoting rapid spreading to new hosts (Russell et al., 2003; Chiel et al., 2009; Oliver et al., 2010). Many studies have revealed phylogenetically closely related symbionts in evolutionarily distant hosts, suggesting that bacteria are horizontally transmitted between diverse insect species (Moran et al., 2005, 2008; Ahmed et al., 2013; Skaljac et al., 2017). The complex horizontal transmission routes include shared plants and parasitoids, resulting in the acquisition of novel ecological traits by the host (Russell et al., 2003; Chiel et al., 2009; Caspi-Fluger et al., 2012; Gehrer and Vorburger, 2012; Gonella et al., 2015; Chrostek et al., 2017).

The genus *Serratia* has spread to diverse habitats and the species in this genus have evolved multiple ecological functions (Petersen and Tisa, 2013). Whereas *S. symbiotica* is one of the most common facultative symbionts of aphids (Manzano-Marín et al., 2012), other *Serratia* species are pathogens associated with humans, insects, nematodes, and plants (Petersen and Tisa, 2013). The ubiquity of the genus is correlated with its ability to produce a large number of extracellular proteins (e.g., proteases, lipases, DNAses, and chitinases) that enable the species to thrive within or in close contact with many hosts (Petersen and Tisa, 2014). There are several classes of bacterial proteases, the most common of which is the metalloproteases (Miyoshi, 2013), and their major physiological role is to degrade environmental proteins for bacterial heterotrophic nutrition (Wu and Chen, 2011).

Although *S. symbiotica* is predominantly a mutualist, it acts as a facultative and protective symbiont in *A. pisum* and the black bean aphid (*Aphis fabae* Scopoli), but it has established co-obligate (nutritional) associations with aphids of the Lachninae subfamily and *B. aphidicola* (Manzano-Marin and Latorre, 2016). *S. symbiotica* provides many benefits but it also imposes costs on *A. pisum* by inhibiting reproduction, development and survival (Laughton et al., 2014; Skaljac et al., 2018). Insects must control their symbiont population in order to ensure the success of both partners, and this is frequently associated with trade-offs between investment in life-history traits and the regulation of symbionts (Login et al., 2011; Laughton et al., 2014).

The vast majority of bacterial symbionts have proven difficult to cultivate in the laboratory due to their lifestyle, gene loss, and dependence on host metabolites (Dale and Moran, 2006; Stewart, 2012). However, several cultivable strains of *S. symbiotica* have recently been isolated from *A. fabae* and the sage aphid (*A. passeriniana* Del Guercio; Sabri et al., 2011; Foray et al., 2014; Grigorescu et al., 2018). These strains are transitional forms between free-living and host-dependent symbiotic bacteria and they provide unique opportunities to study different multi-trophic interactions, such as the tritrophic relationship between symbionts, insects and plants (Foray et al., 2014; Renoz et al., 2017).

Bacterial symbionts frequently play a key role in plant–insect interactions, with important implications for plant defence and plant utilisation by insects (Frago et al., 2012; Sugio et al., 2015; Chrostek et al., 2017). Although the diversity of insect symbionts associated with plants has been investigated in detail, the role of symbiotic bacteria in such interactions is unclear. For example, *Rickettsia* spp. and *Wolbachia* spp. infect the sweet potato whitefly (*Bemisia tabaci* Gennadius) and are horizontally transmitted via the host plant to uninfected peers or even different species (Caspi-Fluger et al., 2012; Li S.J. et al., 2017; Li Y.H. et al., 2017). Furthermore, *Cardinium* spp. are transferred between different phloem-feeding insects via plants carrying the symbiont (Gonella et al., 2015). A common factor in many of these studies is that bacterial symbionts are found in different insect organs, including the salivary glands and stylet, enabling insect hosts to inoculate plant tissues with symbionts. Furthermore, *Wolbachia* spp. and *Rickettsia* spp. associated with *B. tabaci* are viable and persist in reservoir plants for an extended duration, suggesting potential interactions with the plant, such as nutrient uptake (Caspi-Fluger et al., 2012; Chrostek et al., 2017; Li S.J. et al., 2017; Li Y.H. et al., 2017).

Bacterial symbionts are known to help their insect hosts overcome plant defense and adapt to host plants. As a defence mechanism, plants frequently produce inhibitors to destroy proteases secreted by herbivorous insects, thus stopping them from digesting plant proteins (Hansen and Moran, 2014; Sugio et al., 2015; Wielkopolan and Obrepalska-Steplowska, 2016). In turn, insects may produce new protease isoforms that are resistant to plant inhibitors, or they may produce proteases at a higher rate (Wielkopolan and Obrepalska-Steplowska, 2016). Remarkably, gut bacteria in the Western corn rootworm (*Diabrotica virgifera virgifera* LeConte) and the velvet bean caterpillar (*Anticarsia gemmatalis* Hübner) produce additional proteases that help the insects to overcome the protease inhibitors produced by plants (Sugio et al., 2015).

Aphids inject infested plants with saliva containing proteases that digest phloem sap proteins, and these enzymes can be inhibited by the broad-spectrum metalloprotease inhibitor EDTA (Furch et al., 2015). Given that *Serratia* spp. are known to secrete a variety of extracellular enzymes (Hase and Finkelstein, 1993; Renoz et al., 2017), we hypothesise that *S. symbiotica* proteases may help aphids to exploit plants more efficiently by digesting plant proteins. We therefore investigated the localization of *S. symbiotica* in aphid mouthparts and wounded plants, analysed the proteome of *S. symbiotica* cultured in liquid medium to identify secreted proteases, and determined whether the transcripts encoding these enzymes are present in the aphids and also their host plants.

## Materials and Methods

### Aphids and Bacterial Symbionts

#### Maintenance of Aphids and Detection of Symbionts

Parthenogenetic *A. pisum* clone LL01 was reared under controlled conditions on the host plant *Vicia faba* var. *minor* as previously described (Luna-Ramirez et al., 2017; Will et al., 2017). The LL01 clone was obtained from Dr. Torsten Will (Justus-Liebig University, Giessen, Germany) and has been used in our research since 2009. We have previously shown that every individual carries *B. aphidicola* and *S. symbiotica* (Luna-Ramirez et al., 2017; Skaljac et al., 2018). A previously established, *Serratia*-free *A. pisum* line was used as a control, whereas the original (infected) aphid line is described hereafter as *Serratia*-positive (Skaljac et al., 2018). The infection status of these aphid lines was regularly checked to detect any potential contamination, especially the presence of *S. symbiotica* in the *Serratia*-free line.

We detected *S. symbiotica* in aphids and plants by extracting total DNA from *Serratia*-positive or *Serratia*-free aphids and *V. faba* tissues using the CTAB method (Luna-Ramirez et al., 2017). We then used *Serratia*-specific primers to detect *S. symbiotica* 16S rDNA in the aphids and *V. faba* plants by PCR (Supplementary Table S1). Amplicons were eluted using the NucleoSpin Gel and PCR Clean-up kit (Macherey-Nagel, Düren, Germany), and sequenced for verification on a 3730xl DNA analyzer (Macrogen Europe, Amsterdam, Netherlands). The resulting sequences were screened against the NCBI nr database using BLAST. The nucleotide sequences of the *S. symbiotica* 16*S* rDNA identified in this study were deposited in GenBank under accession numbers MH447605–MH447629 (whole aphid body), MH447630 (aphid gut), and MH447631–MH447632 (*V. faba* carrying *S. symbiotica*).

Proteomic analysis was carried out using the cultivable *S. symbiotica* strain CWBI-2.3 (DSM no. 23270), originally isolated from *A. fabae*. This strain was obtained from the Leibniz Institute DSMZ (Braunschweig, Germany) and was cultivated as recommended by the supplier. Briefly, the strain was grown in 535 liquid medium at 28°C overnight in a shaking incubator at 200 rpm. Cells were harvested by centrifugation at 453 × *g* for 30 min at 10°C, and the supernatant was stored at -80°C.

#### Quantification and Visualisation of *S. symbiotica* in *A. pisum* and Its Host Plants

At least three biological replicates of 30 adult *A. pisum* (10 days old) from *Serratia-*positive and *Serratia*-free aphid lines were released into Petri dishes containing *V. faba* discs (2 cm diameter) on 1% agar. After 2 days, aphids were collected in groups of 10 and stored in absolute ethanol at -20°C. Small strips of *V. faba* disc (2 cm × 3 mm) were cut from each replicate immediately after feeding and also 5 and 10 days post-feeding. All insect and plant samples were surface sterilised as previously described (Grigorescu et al., 2018) before DNA or further RNA extraction to ensure that *S. symbiotica* cells and gene expression represented bacteria present inside the tissues.

The abundance of *S. symbiotica* in the *A. pisum* and *V. faba* samples was determined by quantitative PCR (qPCR) as previously described with modifications (Luna-Ramirez et al., 2017). Briefly, genomic DNA was extracted using the CTAB method and a 133-bp fragment of the *S. symbiotica* dnaK gene (Supplementary Table S1) was amplified using the StepOnePlus Real-Time PCR System (Applied Biosystems, Waltham, MA, United States). The 10-μL reaction mixture comprised 2 μL of DNA template (50 ng/μL), 10 μM of each specific primer and 5 μL of Power SYBR Green PCR Master Mix (Applied Biosystems). For each sample, three independent reactions were carried out for each primer pair. The relative abundance of the *dnaK* gene in the *Serratia-*positive and *Serratia*-free aphid lines was determined after normalisation to the *ribosomal protein L32* (*rpl32*) reference gene in aphids (Pfaffl, 2001). Furthermore, the relative abundance of *S. symbiotica* in *V. faba* plants exposed to the two aphid lines was determined after normalisation to the *V. faba* actin reference gene (Supplementary Table S1). Significant differences in abundance were confirmed using Student’s *t*-test in IBM SPSS v23 (Armonk, New York, NY, United States), with statistical significance defined as *p* < 0.05.

We visualised *S. symbiotica* by fluorescence *in situ* hybridization (FISH) in dissected mouthparts, salivary glands and guts of adult aphids as we previously described (Luna-Ramirez et al., 2017). In addition, hand-cut longitudinal stem sections of *V. faba* seedlings that were highly infested with aphids for at least 10 days were analysed by FISH as previously reported (Ghanim et al., 2009). Negative controls consisted of uninfected samples and no-probe staining (Supplementary Figures S1, S2 and Supplementary Table S2). The primers and probe used for the quantification and visualisation of *S. symbiotica* are listed in Supplementary Table S1.

#### Horizontal Transmission of *S. symbiotica* Between *A. pisum* Individuals via Host Plants

To determine whether *S. symbiotica* detected in *V. faba* plants can be acquired by *Serratia-*free aphids, 30 aphids (10 days old) from the *Serratia*-positive line were fed on *V. faba* discs in five replicates for 2 days and then removed (Supplementary Figure S4). Meanwhile, 30 age-synchronised aphids (2 days old) from the *Serratia*-free line were released onto each *V. faba* disc previously occupied by the *Serratia*-positive aphids (Supplementary Figure S3). The *Serratia*-free aphids were allowed to feed for 3 days before transfer to a cage containing non-infested *V. faba* plants. These aphids are described hereafter as *Serratia-*reinfected and were kept in the rearing cage for the next 2 months to ensure the bacterial symbiont could spread among the aphid population.

**FIGURE 1:**
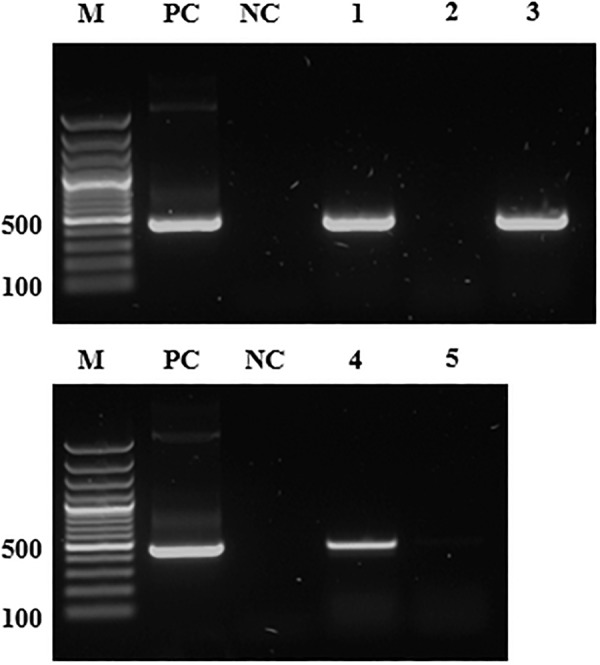
The detection of *S. symbiotica* genomic DNA by PCR. M, DNA marker (size in base pairs); PC, positive control (pGEM T-Easy vector with *S. symbiotica* 16*S* rDNA); NC, negative control (distilled water); lane 1, *Serratia*-positive aphids; lane 2, *Serratia*-free aphids; lane 3, *Serratia*-reinfected aphids (2 months after infection event); lane 4, *V. faba* plant infested with *Serratia*-positive aphids; lane 5, *V. faba* plant infested with *Serratia*-free aphids. The *Serratia* specific primers used for PCR are listed in Supplementary Table S1. Amplicon size ∼480 bp.

The *V. faba* discs, mothers from both aphid lines and their randomly selected offspring were tested by PCR for the presence of *S. symbiotica* (Figure 1). Two months after infection, at least 30 *Serratia-*reinfected aphids were individually tested by PCR to confirm the transmission of *S. symbiotica* (Figure 1 and Supplementary Table S3). The nucleotide sequences of *S. symbiotica* 16*S* rDNA identified in this study were deposited in GenBank under accession numbers MK424314–MK424325 for the *Serratia*-reinfected aphids. The three aphid lines were strictly separated to prevent contamination. However, to avoid false positive transmission results due to potential contamination with the symbiont, we also included a negative control comprising *Serratia*-free aphids as both donors and recipients (Supplementary Table S3).

#### Phylogenetic Analysis of *S. symbiotica*

A phylogenetic tree was constructed using MEGA v7.0 (Kumar et al., 2016). DNA sequence similarities among *Serratia* species were investigated using the BLAST search tool^1^. ClustalW was used for multiple sequence alignments with default parameters. The phylogenetic tree was constructed using the maximum-likelihood method with a Tamura-Nei distance matrix. Bootstrap analysis of 1000 replicates was used to deduce confidence levels. The phylogenetic tree was displayed, manipulated and annotated using iTOL v4.2 (Letunic and Bork, 2016).

### Proteomic Analysis of *S. symbiotica* CWBI-2.3 Culture Medium and Identification of Genes Encoding Proteolytic Enzymes in Aphids and Plants

#### Liquid Chromatography–Mass Spectrometry (LC-MS)

The concentrated supernatant of *S. symbiotica* CWBI-2.3 cells in 535 medium was fractionated by sodium dodecyl sulfate polyacrylamide gel electrophoresis (SDS-PAGE) in 16.5% tricine gradient gels (BioRad, Munich, Germany). The protein bands were stained with Coomassie Brilliant Blue and excised from the gel matrix for tryptic digestion as previously described (Shevchenko et al., 2006). For LC-MS analysis, samples were reconstituted in 50 μL aqueous 1% formic acid and 1 μL of the peptide mixture was injected into a UPLC M-class system (Waters, Eschborn, Germany) coupled online to a Synapt G2-si mass spectrometer equipped with a T-WAVE-IMS device (Waters). Data were acquired in data-dependent acquisition (DDA) and data-independent acquisition (DIA) modes, the latter described as enhanced MS^E^. DIA analysis was supported by ion mobility separation, i.e., high-definition enhanced MS^E^ (HDMS^E^) analysis (Distler et al., 2016).

#### Data Processing and Protein Identification

DDA raw data were first searched against a small database containing common contaminants to remove them (ProteinLynx Global Server v2.5.2, Waters). Remaining spectra were interpreted *de novo* to yield peptide sequences and used as queries for homology-based searching with MS-BLAST (Shevchenko et al., 2001) installed on a local server. MS-BLAST searches were performed against the NCBI nr database and a refined *S. symbiotica* database generated by the *in silico* translation of predicted *S. symbiotica* genes. In parallel, MS/MS spectra were searched against the NCBI nr database combined with the refined *S. symbiotica* database using MASCOT v2.5.1. HDMS^E^ data were searched against the refined *S. symbiotica* protein database and a database containing common contaminants (human keratins and trypsin).

#### Identification and Expression Analysis of *S. symbiotica* Protease Genes in Aphids and Plants

Proteolytic enzymes detected in the supernatant of the *S. symbiotica* CWBI-2.3 strain (Supplementary Table S4) allowed the analysis of the corresponding genes in *S. symbiotica* infecting *A. pisum* and its infested host plants. Complementary DNA (cDNA) sequences for most of the *S. symbiotica* proteases were identified using the Ensembl Bacteria browser^2^ or NCBI databases^3^. Gene-specific PCR primers were designed using Primer3 v4.1.0^4^ to amplify specific regions of the transcribed cDNAs (Koressaar and Remm, 2007; Supplementary Table S1).

Total RNA was extracted from the previously described samples, i.e., aphids from *Serratia*-positive and *Serratia*-free lines, *V. faba* containing or lacking the symbiont, and *S. symbiotica* CWBI-2.3, using the Direct-zol RNA MiniPrep Plus Kit (Zymo Research, Freiburg, Germany). RNA (100 ng) was transcribed using the RevertAid First Strand cDNA synthesis kit (Thermo Fisher Scientific, Dreieich, Germany) to obtain first-strand cDNA. Amplicons from *V. faba* samples infested with *Serratia-*positive aphids were re-amplified because the quantity was low, and were cloned (Supplementary Figures S5, S6) before sequencing together with amplicons from the *Serratia-*positive aphids and the supernatant of *S. symbiotica* CWBI-2.3. Cloning and sequencing were carried out as previously described (Skaljac et al., 2018). Accession numbers for the *S. symbiotica* protease genes are listed in Table 1. The sequences were used to design qRT-PCR primers (Supplementary Table S1) in PrimerQuest (Integrated DNA Technologies, Coralville, IA, United States^5^). Control samples (*Serratia*-free aphids and their host plants, as well as non-infested *V. faba* plants), were negative for the expression of *S. symbiotica* protease genes. *S. symbiotica* CWBI-2.3 cDNA was used as a positive control (Supplementary Figure S5).

**Table 1:** Overview of the genes encoding proteolytic enzymes with associated GenBank accession numbers from *S. symbiotica* expressed in *A. pisum* and its host plant *V. faba* (for additional explanations, see Results section “Proteolytic enzymes associated with *S. symbiotica*”).

Protein identification from supernatant of *S. symbiotica* CWBI-2.3, with GenBank accession number for top-scoring protein of *S. symbiotica*	Samples with identified mRNA from *S. symbiotica* including GenBank accession numbers obtained in this study			Potential molecular function and biological process of a protein^§^
	*Serratia*-positive aphid line	*V. faba* carrying *S. symbiotica*	Culture of *S. symbiotica* CWBI-2.3	
Serine endopeptidase (*DegP*) CDS55594.1	MH458199	nd	MH458200	Hydrolase and protease activity; involved in stress response
Serine endopeptidase (*DegQ*) CDS55928.1	MH458201-MH458202		nd	
Putative IgA-specific serine endopeptidase CDS57070.1	nd	nd	nd	nd
Zn-dependent endopeptidase (*HtpX*) CDS58211.1	MH458203-MH458214			Metalloendopeptidase activity; involved in stress response
Putative M48 family peptidase (*YfgC*) CDS57423.1	MH458227-MH458232			
Putative peptidase (*SohB*) CDS58397.1	MH458196-MH458198; MH458233			Serine-type endopeptidase activity; proteolysis
Peptidase D (*PepD*) CDS55732.1	MH458218	nd	MH458219	Metallopeptidase (Zn peptidase like) activity
Aminopeptidase A (*PepA*) CDS56273.1	MH458215-MH458217			Aminopeptidase (metallopeptidase) activity; proteolysis
Aminopeptidase N (*PepN*) CDS57483.1	MH458220-MH458222	nd	MH458223-MH458226	Aminopeptidase (metallopeptidase) activity

*nd – not determined; ^§^Molecular function and biological process suggested by https://www.uniprot.org; https://www.ebi.ac.uk/interpro/; https://www.ncbi.nlm.nih.gov.*

The *S. symbiotica* genes previously shown to be expressed in *V. faba* carrying *S. symbiotica* (*DegQ, HtpX, YfgC, SohB*, and *PepA*) were chosen for further expression analysis by qRT-PCR because they may be important for tritrophic interactions between symbionts, insects and plants (Table 1). The expression of the five selected genes in *Serratia*-free and *Serratia*-positive aphids was evaluated by qRT-PCR after normalisation to the expression level of the *rpl32* reference gene (Pfaffl, 2001). For each sample, three independent reactions were carried out for each primer pair. The qPCR protocol described above was modified so that the cDNA template was diluted 1:2 with RNase-free water before qRT-PCR (2 μL in a total volume of 10 μL). The relevant target genes and primers are listed in Table 1 and Supplementary Table S1. Data were analysed as described above.

## Results

### *S. symbiotica* in *A. pisum* and Its Host Plants

#### Detection and Visualisation of *S. symbiotica*

Polymerase chain reaction analysis showed that *S. symbiotica* was present in every individual of the *Serratia-*positive line, in multiple tissues including the salivary glands and gut (Supplementary Table S2) confirming findings from our previous study (Skaljac et al., 2018). We found no evidence of the symbiont in the *Serratia-*free line over many generations of rearing under laboratory conditions (Figure 1). Furthermore, the same PCR also showed that *S. symbiotica* was present in *V. faba* plants infested with *Serratia-*positive aphids, whereas no symbionts were detected in the plants exposed to the *Serratia*-free aphid line (Figure 1).

**FIGURE 2:**
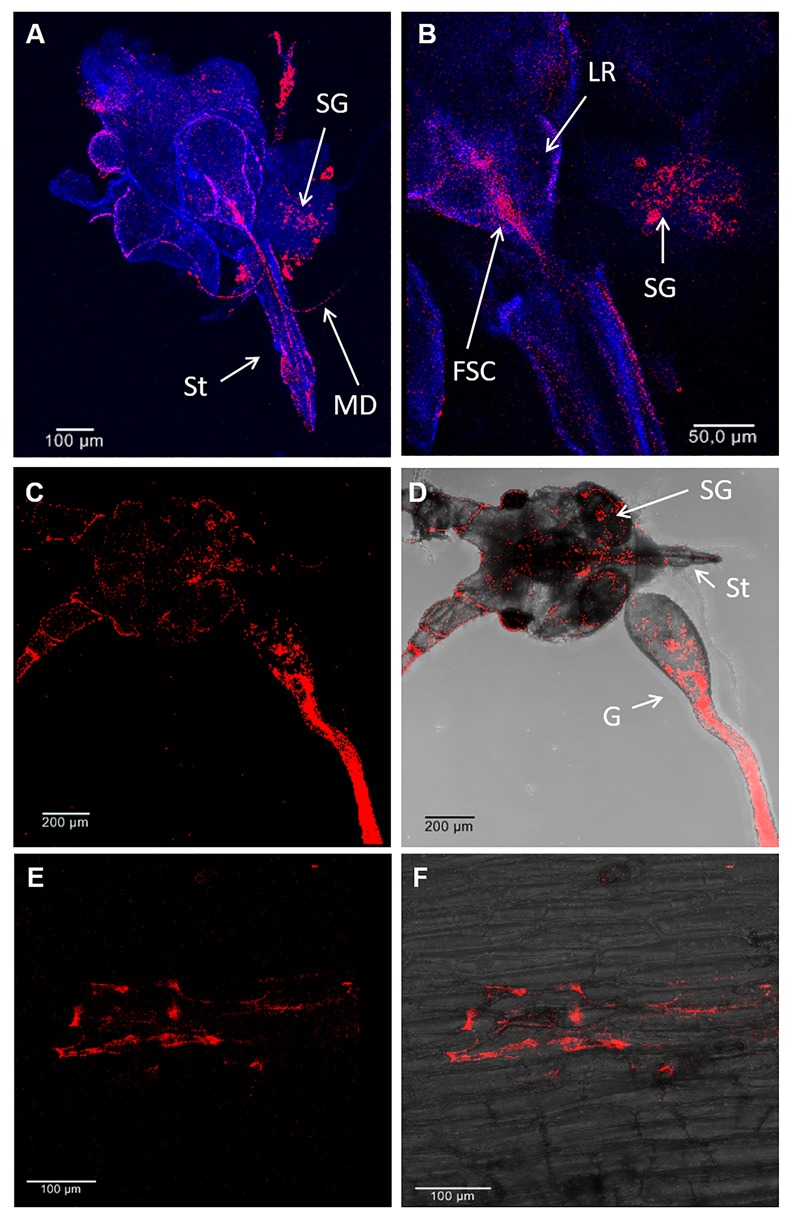
Localization of *S. symbiotica* by fluorescence *in situ* hybridization (FISH) in *A. pisum* mouthparts and *V. faba* tissues. Detection of *S. symbiotica* (red) in the head (mouthparts, salivary glands and gut) of a 10-day-old adult aphids **(A–D)** and *V. faba* longitudinal stem sections under dark field **(E)** and bright field **(F)** imaging. Nuclei were counterstained with DAPI (dark blue). Abbreviations: MD, mandible; SG, salivary gland; St, stylet; LR, labrum; FSC, food and salivary canal; G, gut.

Fluorescence *in situ* hybridization analysis with a probe specific for *S. symbiotica* was used to confirm the PCR data (Supplementary Table S2) and to reveal the distribution of *S. symbiotica* within aphid and *V. faba* tissues. The *S. symbiotica* signal was abundant in the aphid gut (Figure 2C,D), but also in salivary glands and associated mouthparts (stylet, mandibles, labrum, food, and salivary canal) (Figure 2A–D). At this resolution, we were unable to determine whether *S. symbiotica* was present in one or both canals, but in either case our results indicated its route into aphids with the phloem sap or outward with the saliva. We also observed *S. symbiotica* cells in *V. faba* tissues wounded by the penetrating stylet (Figure 2E,F). The symbiont was not detected in non-infested host plants or those infested with the *Serratia*-free line.

**FIGURE 3:**
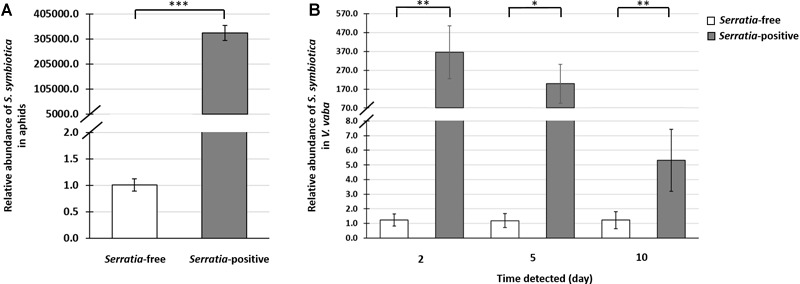
Quantitative PCR analysis of *S. symbiotica* in *A. pisum* and *V. faba.* Data show the relative abundance of the *S. symbiotica dnaK* gene compared to the *rpl32* reference gene in aphids and the *actin* reference gene in plants. This was used to determine the abundance of *S. symbiotica* in the *Serratia*-positive and *Serratia-*free aphid lines **(A)**, and in *V. faba* leaves after exposure to each aphid line, after retention times of 2, 5, and 10 days **(B)**. Statistical significance is indicated as follows: ^∗^*p* < 0.05, ^∗∗^*p* < 0.01, ^∗∗∗^*p* < 0.001.

Quantification by qPCR revealed that *S. symbiotica* was remarkably abundant in *Serratia*-positive aphids (Supplementary Table S5 and Figure 3A). Furthermore, we detected large numbers of *S. symbiotica* in *V. faba* plants after exposure to aphids from the *Serratia*-positive line for 2 days. When the aphids were removed from the host plants, the numbers of *S. symbiotica* fell progressively at the subsequent testing points, 5 and 10 days post-feeding (Figure 3B and Supplementary Table S5). However, *S. symbiotica* was still significantly more abundant in these plants, even 10 days post-feeding, compared to plants exposed to aphids from the *Serratia*-free line (Figure 3B and Supplementary Table S5).

#### Phylogenetic Placement of *S. symbiotica*

Our phylogenetic analysis of *S. symbiotica* incorporated 28 partial 16*S* rDNA sequences derived from the analysis of *A. pisum* and *V. faba* specimens. These sequences were compared with reference sequences from GenBank. *S. symbiotica* from the aphids and *V. faba* plants in this study clustered together with *S. symbiotica* CWBI-2.3 isolated from *A. fabae*, but also with most of the *S. symbiotica* sequences identified in other clones of *A. pisum* (Supplementary Figure S4).

#### Horizontal Transmission of *S. symbiotica* in Aphids via Host Plants

The detection of *S. symbiotica* in the mouthparts of *Serratia*-positive aphids and wounded plant tissues exposed to these aphids led us to investigate whether this symbiont was transmitted to naïve aphids after feeding on *V. faba* plants containing the bacteria. When *V. faba* discs were exposed to *Serratia*-positive aphids for 2 days, the bacterial symbiont was detected by PCR in all plant samples (Figure 1). Sequences from *S. symbiotica* detected in the plant were identical to those in the *Serratia*-positive aphids (Supplementary Figure S4). Releasing *Serratia*-free aphids to feed on plant discs carrying the symbiont for 3 days enabled the transmission of the symbiont to naïve aphids. This was confirmed by PCR analysis and sequencing 2 months after the infection event (Figure 1 and Supplementary Table S3). The incubation period of 2 months enabled *S. symbiotica* to spread among all formerly *Serratia*-free aphids, thus increasing the likelihood of inducing the previously observed biological effects and fitness costs (Skaljac et al., 2018). We did not detect *S. symbiotica* following the exposure of *V. faba* to *Serratia*-free aphids (Figure 1). During our experiments, no symptoms of bacterial disease were observed in *V. faba* infested with *Serratia*-positive aphids, indicating that the symbiont is not phytopathogenic in nature.

### Proteolytic Enzymes Associated With *S. symbiotica*

#### Identification of Proteolytic Enzymes Released by *S. symbiotica* CWBI-2.3

Sodium dodecyl sulfate polyacrylamide gel electrophoresis analysis of the *S. symbiotica* CWBI-2.3 culture supernatant revealed a remarkable number of potentially secreted proteins (Supplementary Figure S7). In total, 246 different extracellular proteins were identified by LC-MS/MS and characterised, representing numerous categories of biological processes (Supplementary Table S6). Among these proteins, we identified 15 enzymes with predicted proteolytic activity, including metalloproteases (Supplementary Table S4). These enzymes potentially facilitate the degradation of host plant proteins as their annotations suggest^6^^,^^7^^,^^8^. In total, nine *S. symbiotica* proteases with complete genomic information were included for further analysis (Table 1): the serine endopeptidases DegP and DegQ, the putative IgA-specific Zn-dependent serine endopeptidase HtpX, the putative M48 family peptidase YfgC, the putative peptidase SohB, peptidase D (PepD), aminopeptidase A (PepA) and aminopeptidase N (PepN).

#### *S. symbiotica* Genes Encoding Proteolytic Enzymes in *A. pisum* and Its Host Plants

**FIGURE 4:**
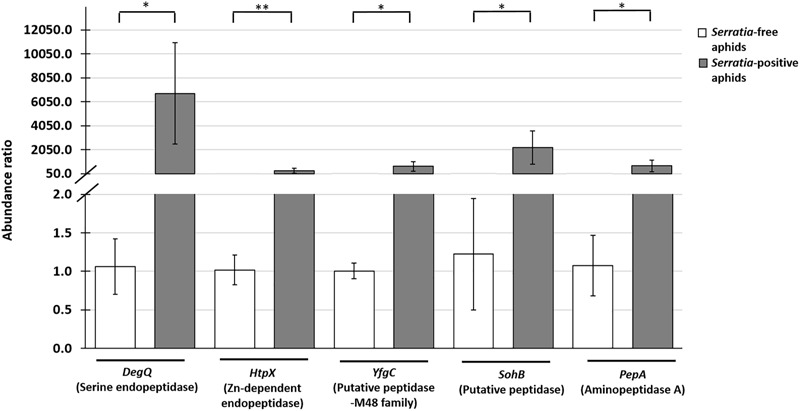
Quantitative RT-PCR analysis showing the relative expression of five *S. symbiotica* genes (*DegQ, HtpX, YfgC, SohB*, and *PepA*) encoding proteolytic enzymes associated with the host plant (Table 1) in *Serratia*-positive and *Serratia*-free aphids. The expression data were normalised to the aphid reference gene *rpl32*. Statistical significance is indicated as follows: ^∗^*p* < 0.05, ^∗∗^*p* < 0.01.

Having identified nine *S. symbiotica* CWBI-2.3 extracellular proteases for further analysis, we tested different aphid and plant samples for the presence of the corresponding transcripts. The *DegP, DeqQ, HtpX, YfgC, SohB, PepD, PepA*, and *PepN* transcripts were detected in *Serratia*-positive aphids (Table 1). Furthermore, the *DegQ, HtpX, YfgC, SohB*, and *PepA* transcripts were also present (albeit at much lower levels) in plants previously exposed to the *Serratia*-positive aphids (Table 1 and Supplementary Figure S5). The *DegQ, HtpX, YfgC, SohB*, and *PepA* transcripts representing serine endopeptidases and metallopeptidases were selected for further qRT-PCR analysis because they may be relevant in the context of aphid–plant interactions. Quantitative RT-PCR analysis revealed that these five genes were more strongly expressed in *Serratia*-positive aphids than *Serratia*-free aphids (Supplementary Table S5 and Figure 4). The same transcripts were below the level of detection in *V. faba* tissues previously infested with *Serratia*-positive aphids (Supplementary Figure S5).

## Discussion

Previous studies have shown that *S. symbiotica* colonises several *A. pisum* tissues, specifically the bacteriocytes, gut and haemolymph (Moran et al., 2005; Sabri et al., 2013; Luna-Ramirez et al., 2017; Skaljac et al., 2018). The experiments described here allow us to expand that distribution to include the aphid salivary glands and associated mouthparts (Figure 2A–D). Furthermore, *S. symbiotica* was detected in the stylet and in wounded plant tissues, providing strong evidence that aphids inoculate host plants with their bacterial symbionts (Figure 2E,F). In agreement with our data, recent studies of bacterial symbionts (e.g., *Rickettsia* spp., *Wolbachia* spp., and *Cardinium* spp.) associated with herbivorous insects (e.g., *B. tabaci* or *Scaphoideus titanus* Ball) reported that bacteria found in the feeding apparatus and gut were also observed in the host plants (Skaljac et al., 2010; Brumin et al., 2012; Caspi-Fluger et al., 2012; Chrostek et al., 2017; Li S.J. et al., 2017; Li Y.H. et al., 2017). The localization of cultivable strains of *S. symbiotica* (e.g., CWBI-2.3) associated mainly with *Aphis* species is currently thought to be limited to the gut, with no cells detected in the haemolymph (Pons et al., 2019). *S. symbiotica* CWBI-2.3 is able to colonise the entire *A. pisum* gut within just a few days after artificial infection via a specialised diet, without triggering an immune response or affecting survival (Renoz et al., 2015). It would be interesting to determine whether non-cultivable *S. symbiotica* strains are localised differently in the *A. pisum* as previously shown for *Rickettsia* spp. in *B. tabaci* (Gottlieb et al., 2008; Caspi-Fluger et al., 2011). We detected *S. symbiotica* in many *A. pisum* tissues (Figure 2D), including the bacteriome and ovarioles, whereas a more restricted distribution was reported in earlier studies (Moran et al., 2005; Luna-Ramirez et al., 2017).

In Israeli populations of *B. tabaci, Rickettsia* spp. displayed a “scattered” distribution, in which the symbiont was present in the haemocoel, excluding the bacteriocytes, or a “confined” distribution, in which it was restricted to bacteriocytes (Caspi-Fluger et al., 2011). In contrast, we previously reported that *Rickettsia* spp. are distributed in all *B. tabaci* tissues, including both the haemocoel and bacteriocytes (Skaljac et al., 2010). The *Rickettsia* strains with different localization patterns often featured identical sequences, suggesting they are closely related (Caspi-Fluger et al., 2011). However, even the same symbionts can show different localization patterns and fulfil diverse functions in their insect hosts, depending on the environmental conditions (Gottlieb et al., 2008; Caspi-Fluger et al., 2011).

Our results revealed the remarkable abundance of *S. symbiotica* in *V. faba* plants after only 2 days of exposure to *Serratia-*positive aphids (Figure 3B). When the aphids were removed from the feeding site, the *S. symbiotica* load decreased over the subsequent 10 days (Supplementary Table S5). A similar decline in the number of whitefly-associated *Rickettsia* spp. was reported in cotton leaves (Li Y.H. et al., 2017), suggesting that the production of chemical defence compounds in plants may correlate with the decline of symbionts in plant tissues. In addition to the retention time of *S. symbiotica* in *V. faba*, the viability of symbionts in plant tissues is another key requirement for successful interactions with either the plant or naïve insects (Chrostek et al., 2017). The detection of *S. symbiotica* mRNAs in *V. faba* tissues revealed that the symbiont remains alive and transcriptionally active in the plant (Table 1). This was previously shown in the *Rickettsia* and *Wolbachia* symbionts of *B. tabaci* (Caspi-Fluger et al., 2012; Li S.J. et al., 2017; Li Y.H. et al., 2017). Future studies should include experiments to determine whether *S. symbiotica* is able to multiply in the host plants as previously described for phytopathogenic *S. marcescens* (Petersen and Tisa, 2013).

The transmission of symbionts via host plants can have a significant impact on the ecology and evolution on both the symbiont and its insect host (Chrostek et al., 2017). For instance, *Rickettsia* spp. has rapidly spread among populations of *B. tabaci* across the southwestern United States, significantly affecting life-history traits by accelerating development, promoting survival into adulthood, and encouraging the production of more offspring (Himler et al., 2011). At the same time, the transmission of *Rickettsia* spp. via plants may have favoured the rapid spreading of this symbiont among populations of *B. tabaci* (Caspi-Fluger et al., 2012). Symbionts help herbivorous insects to utilise plants (e.g., the gut bacteria in *D. virgifera virgifera*), whereas other bacteria have evolved from arthropod symbionts into insect-vectored plant pathogens (e.g., *Arsenophonus* spp.; Sugio et al., 2015; Chrostek et al., 2017). This shows the complexity of the interactions between insects, their symbionts and plants in response to different selection pressures (Shah and Walling, 2017).

We investigated the possibility that *S. symbiotica* was transmitted to uninfected aphids via the host plant, as previously shown for other insect–symbiont systems (Chrostek et al., 2017). Accordingly, we found that when *V. faba* plants containing *S. symbiotica* were fed to uninfected aphids, the plants acted as reservoirs for the efficient transmission of symbionts, resulting in the reinfection of all exposed individuals (Figure 1 and Supplementary Table S3). Several studies have indicated that symbionts of herbivorous insects can be transmitted via honeydew (Darby and Douglas, 2003; Chrostek et al., 2017; Pons et al., 2019). We previously detected *S. symbiotica* in the honeydew of *Serratia*-positive *A. pisum*, so this transmission route cannot be ruled out in natural environments (Skaljac et al., 2018). The transmission route of cultivable *S. symbiotica* strains (e.g., CWBI-2.3) is unknown in *Aphis* species, but this study provides important clues to support the plant reservoir hypothesis. Bacterial symbionts are transmitted maternally with high fidelity. We previously detected *S. symbiotica* in the bacteriomes and ovarioles of *A. pisum* suggesting that this symbiont probably spreads via both horizontal and vertical transmission (Luna-Ramirez et al., 2017).

Given that *S. symbiotica* is one of the most common symbionts of aphids and that *Serratia* species can secrete extracellular enzymes to fulfil their roles in diverse ecological niches, we propose that some of the proteins secreted by *S. symbiotica* (especially proteolytic enzymes) might help the aphids to exploit their host plants more efficiently (Manzano-Marín et al., 2012; Petersen and Tisa, 2013; Sugio et al., 2015; Renoz et al., 2017). In order to test this hypothesis, we used the cultivable *S. symbiotica* strain CWBI-2.3 to identify extracellular proteases and investigate the abundance of the corresponding transcripts in aphids and *V. faba* plants. Our proteomic analysis of the *S. symbiotica* CWBI-2.3 culture supernatant revealed a diverse spectrum of secreted proteins, in agreement with the recently published membrane and cytosolic proteome of this species (Renoz et al., 2017; Supplementary Tables S4, S6). Our study has expanded the spectrum of *S. symbiotica* proteolytic enzymes (Renoz et al., 2017) to include serine endopeptidases (DegP and DegQ), M48 family metallopeptidases (HtpX and YfgC), aminopeptidases (PepA and PepN) and the other peptidases listed in Supplementary Table S4. Proteases are well-known virulence factors in pathogenic *Serratia* species (Petersen and Tisa, 2014) and they play important roles in the degradation of tissues that allow *Serratia* spp. to survive and proliferate within the host (Matsumoto, 2004).

The proteomic analysis of candidate *S. symbiotica* proteases in host plant tissues is not feasible due to the competition from endogenous plant proteins, so we focused on the highly sensitive detection of the corresponding transcripts. Most of the *S. symbiotica* CWBI-2.3 genes encoding proteases in the culture medium were also detected in both *Serratia*-positive aphids and in plants containing symbiont cells (Table 1). The *S. symbiotica* protease genes identified in *V. faba* were strongly expressed in *Serratia*-positive aphids (Figure 4 and Supplementary Table S5), suggesting that *S. symbiotica* may indeed help aphids to digest phloem sap proteins and potentially to resist protease inhibitors (Zhu-Salzman and Zeng, 2015). Several studies have highlighted the importance of symbiotic bacteria in the ability of insects to exploit host plants more efficiently by suppressing plant defence mechanisms and/or by expanding the host plant range. For example, this has been shown for *B. tabaci* and its symbiont *H. defensa*, and in the Colorado potato beetle (*Leptinotarsa decemlineata* Say) and its symbionts representing the bacterial genera *Stenotrophomonas, Pseudomonas*, and *Enterobacter* (Frago et al., 2012; Su et al., 2015; Sugio et al., 2015; Chung et al., 2017).

In this study, transcripts encoding candidate proteases were present at very low levels in plants previously infested with *Serratia*-positive aphids (Supplementary Figure S5). This suggests that the detection of transcripts in *V. faba* is most likely associated with the presence of the symbiont (Table 1). On the other hand, the abundance of *S. symbiotica* in aphid tissues (Figure 2A–D, 3A) together with the strong expression of protease genes associated with *Serratia*-positive aphids (Figure 4) suggest that the proteases may be active in the aphid gut and salivary glands but not necessarily in the host plant. These assumptions are supported by previous studies showing that plant-derived protease inhibitors inactivate digestive enzymes in the insect gut, preventing the digestion and absorption of nutrients (Ryan, 1990; Hansen and Moran, 2014). Therefore, *S. symbiotica* proteases are more likely to fulfil their role in the aphid gut (or salivary glands) rather than the host plants.

In summary, we investigated the localization of *S. symbiotica* in aphid mouthparts and host plant tissues and confirmed the transmission of this symbiont via plants, potentially explaining its high frequency among aphid populations. We expanded the repertoire of proteolytic enzymes produced by *S. symbiotica* in liquid medium and confirmed the strong expression of the corresponding genes in aphids and their weaker expression in infested host plants. We conclude that plants serve as reservoirs for the transmission of protease-secreting bacterial symbionts among aphids, suggesting that such symbionts could be important mediators of aphid–plant interactions. Investigating the precise nature of the symbiotic relationship described in this study will help to determine whether *S. symbiotica* uses proteases to spread among insect hosts, while in return enabling the insect to exploit plants more efficiently by the suppression of protease inhibitors.

There may be ecological and genomic differences between the two *S. symbiotica* strains used in this study, and accordingly some of the symbiotic proteases originating from the uncultivable strain may have been overlooked. Therefore, future studies should investigate extracellular proteases originating from different *S. symbiotica* strains released under diverse ecological conditions (e.g., exposure to a range of host plants). Furthermore, it would be interesting to determine the precise functions of the proteases listed in Table 1 to see whether any of them are specifically involved in the suppression of plant defences, the digestion of plant proteins or the proliferation of the symbiont. It would also be valuable to compare defence mechanisms in plants attacked by *Serratia*-positive and *Serratia*-free aphids because this symbiont may have the potential to evolve into a plant pathogen that uses aphids as vectors.

## Author Contributions

MS, HV, NW, and SM contributed to the study design, carried out the molecular laboratory work, analysed the data, and drafted the manuscript. AV conceived, designed, and coordinated the study, and helped draft the manuscript. All authors agreed to be accountable for the content of the article and give approval for its publication.

## Conflict of Interest Statement

The authors declare that the research was conducted in the absence of any commercial or financial relationships that could be construed as a potential conflict of interest.

## Footnotes

1 http://blast.ncbi.nlm.nih.gov/Blast.cgi

2 http://bacteria.ensembl.org/index.html

3 https://www.ncbi.nlm.nih.gov/

4 http://primer3.ut.ee/

5 http://eu.idtdna.com/PrimerQuest

6 https://www.uniprot.org

7 https://www.ebi.ac.uk/interpro/

8 https://www.ncbi.nlm.nih.gov

## References

[B1] Ahmed M. Z. De Barro P. J. Ren S. X. Greeff J. M. Qiu B. L. (2013). Evidence for horizontal transmission of secondary endosymbionts in the *Bemisia tabaci* cryptic species complex. *PLoS One* 8:e53084. 10.1371/journal.pone.0053084 23308142 PMC3538644

[B2] Brumin M. Levy M. Ghanim M. (2012). Transovarial transmission of Rickettsia spp. and organ-specific infection of the whitefly *Bemisia tabaci*. *Appl. Environ. Microbiol.* 78 5565–5574. 10.1128/AEM.01184-12 22660706 PMC3406125

[B3] Caspi-Fluger A. Inbar M. Mozes-Daube N. Katzir N. Portnoy V. Belausov E. (2012). Horizontal transmission of the insect symbiont Rickettsia is plant-mediated. *Proc. Biol. Sci.* 279 1791–1796. 10.1098/rspb.2011.2095 22113034 PMC3297456

[B4] Caspi-Fluger A. Inbar M. Mozes-Daube N. Mouton L. Hunter M. S. Zchori-Fein E. (2011). Rickettsia ‘in’ and ‘out’: two different localization patterns of a bacterial symbiont in the same insect species. *PLoS One* 6:e21096. 10.1371/journal.pone.0021096 21712994 PMC3119683

[B5] Chiel E. Inbar M. Gottlieb Y. Kelly S. E. Asplen M. K. Hunter M. S. (2009). Almost there: transmission routes of bacterial symbionts between trophic levels. *PLoS One* 4:e4767. 10.1371/journal.pone.0004767 19274091 PMC2651630

[B6] Chrostek E. Pelz-Stelinski K. Hurst G. D. D. Hughes G. L. (2017). Horizontal transmission of intracellular insect symbionts via plants. *Front. Microbiol.* 8:2237. 10.3389/fmicb.2017.02237 29234308 PMC5712413

[B7] Chung S. H. Scully E. D. Peiffer M. Geib S. M. Rosa C. Hoover K. (2017). Host plant species determines symbiotic bacterial community mediating suppression of plant defenses. *Sci. Rep.* 7:39690. 10.1038/srep39690 28045052 PMC5206732

[B8] Consortium I. A. G. (2010). Genome sequence of the pea aphid *Acyrthosiphon pisum*. *PLoS Biol.* 8:e1000313. 10.1371/journal.pbio.1000313 20186266 PMC2826372

[B9] Dale C. Moran N. A. (2006). Molecular interactions between bacterial symbionts and their hosts. *Cell* 126 453–465. 10.1016/j.cell.2006.07.014 16901780

[B10] Darby A. C. Douglas A. E. (2003). Elucidation of the transmission patterns of an insect-borne bacterium. *Appl. Environ. Microbiol.* 69 4403–4407. 10.1128/AEM.69.8.4403-4407.2003 12902222 PMC169131

[B11] Distler U. Kuharev J. Navarro P. Tenzer S. (2016). Label-free quantification in ion mobility-enhanced data-independent acquisition proteomics. *Nat. Protoc.* 11 795–812. 10.1038/nprot.2016.042 27010757

[B12] Foray V. Grigorescu A. S. Sabri A. Haubruge E. Lognay G. Francis F. (2014). Whole-genome sequence of serratia symbiotica strain CWBI-2.3T, a free-living symbiont of the black bean aphid *Aphis fabae*. *Genome Announc.* 2:e00767-14. 10.1128/genomeA.00767-14 25146134 PMC4153493

[B13] Frago E. Dicke M. Godfray H. C. (2012). Insect symbionts as hidden players in insect-plant interactions. *Trends Ecol. Evol.* 27 705–711. 10.1016/j.tree.2012.08.013 22985943

[B14] Furch A. C. van Bel A. J. Will T. (2015). Aphid salivary proteases are capable of degrading sieve-tube proteins. *J. Exp. Bot.* 66 533–539. 10.1093/jxb/eru487 25540441

[B15] Gehrer L. Vorburger C. (2012). Parasitoids as vectors of facultative bacterial endosymbionts in aphids. *Biol. Lett.* 8:613. 10.1098/rsbl.2012.0144 22417790 PMC3391472

[B16] Ghanim M. Brumin M. Popovski S. (2009). A simple, rapid and inexpensive method for localization of tomato yellow leaf curl virus and *Potato leafroll* virus in plant and insect vectors. *J. Virol. Methods* 159 311–314. 10.1016/j.jviromet.2009.04.017 19406154

[B17] Gonella E. Pajoro M. Marzorati M. Crotti E. Mandrioli M. Pontini M. (2015). Plant-mediated interspecific horizontal transmission of an intracellular symbiont in insects. *Sci. Rep.* 5:15811. 10.1038/srep15811 26563507 PMC4643326

[B18] Gottlieb Y. Ghanim M. Gueguen G. Kontsedalov S. Vavre F. Fleury F. (2008). Inherited intracellular ecosystem: symbiotic bacteria share bacteriocytes in whiteflies. *FASEB J.* 22 2591–2599. 10.1096/fj.07-101162 18285399

[B19] Grigorescu A. S. Renoz F. Sabri A. Foray V. Hance T. Thonart P. (2018). Accessing the hidden microbial diversity of aphids: an illustration of how culture-dependent methods can be used to decipher the insect microbiota. *Microb. Ecol.* 75 1035–1048. 10.1007/s00248-017-1092-x 29119316

[B20] Hansen A. K. Moran N. A. (2011). Aphid genome expression reveals host–symbiont cooperation in the production of amino acids. *Proc. Natl. Acad. Sci. U.S.A.* 108 2849–2854. 10.1073/pnas.1013465108 21282658 PMC3041126

[B21] Hansen A. K. Moran N. A. (2014). The impact of microbial symbionts on host plant utilization by herbivorous insects. *Mol. Ecol.* 23 1473–1496. 10.1111/mec.12421 23952067

[B22] Hase C. C. Finkelstein R. A. (1993). Bacterial extracellular zinc-containing metalloproteases. *Microbiol. Rev.* 57 823–837. 8302217 10.1128/mr.57.4.823-837.1993 PMC372940

[B23] Himler A. G. Bergen J. E. Kozuch A. Kelly S. E. Tabashnik B. E. Chiel E. (2011). Rapid spread of a bacterial symbiont in an invasive whitefly is driven by fitness benefits and female bias. *Science* 332:254. 10.1126/science.1199410 21474763

[B24] Koressaar T. Remm M. (2007). Enhancements and modifications of primer design program Primer3. *Bioinformatics* 23 1289–1291. 10.1093/bioinformatics/btm091 17379693

[B25] Kumar S. Stecher G. Tamura K. (2016). MEGA7: molecular evolutionary genetics analysis version 7.0 for Bigger Datasets. *Mol. Biol. Evol.* 33 1870–1874. 10.1093/molbev/msw054 27004904 PMC8210823

[B26] Laughton A. M. Fan M. H. Gerardo N. M. (2014). The combined effects of bacterial symbionts and aging on life history traits in the pea aphid, *Acyrthosiphon pisum*. *Appl. Environ. Microbiol.* 80 470–477. 10.1128/AEM.02657-13 24185857 PMC3911086

[B27] Letunic I. Bork P. (2016). Interactive tree of life (iTOL) v3: an online tool for the display and annotation of phylogenetic and other trees. *Nucleic Acids Res.* 44 W242–W245. 10.1093/nar/gkw290 27095192 PMC4987883

[B28] Li S. J. Ahmed M. Z. Lv N. Shi P. Q. Wang X. M. Huang J. L. (2017). Plant-mediated horizontal transmission of *Wolbachia* between whiteflies. *ISME J.* 11 1019–1028. 10.1038/ismej.2016.164 27935594 PMC5364347

[B29] Li Y. H. Ahmed M. Z. Li S. J. Lv N. Shi P. Q. Chen X. S. (2017). Plant-mediated horizontal transmission of Rickettsia endosymbiont between different whitefly species. *FEMS Microbiol. Ecol.* 93:fix138. 10.1093/femsec/fix138 29069333

[B30] Login F. H. Balmand S. Vallier A. Vigneron A. Rochat D. Heddi A. (2011). Antimicrobial peptides keep insect endosymbionts under control. *Science* 334 362–365. 10.1126/science.1209728 22021855

[B31] Luna-Ramirez K. Skaljac M. Grotmann J. Kirfel P. Vilcinskas A. (2017). Orally delivered scorpion antimicrobial peptides exhibit activity against pea aphid (*Acyrthosiphon pisum*) and its bacterial symbionts. *Toxins* 9:E261. 10.3390/toxins9090261 28837113 PMC5618194

[B32] Manzano-Marín A. Lamelas A. Moya A. Latorre A. (2012). Comparative genomics of *Serratia* spp.: two paths towards endosymbiotic life. *PLoS One* 7:e47274. 10.1371/journal.pone.0047274 23077583 PMC3471834

[B33] Manzano-Marin A. Latorre A. (2016). Snapshots of a shrinking partner: genome reduction in *Serratia symbiotica*. *Sci. Rep.* 6:32590. 10.1038/srep32590 27599759 PMC5013485

[B34] Matsumoto K. (2004). Role of bacterial proteases in pseudomonal and serratial keratitis. *Biol. Chem.* 385 1007–1016. 10.1515/BC.2004.131 15576320

[B35] Miyoshi S. I. (2013). Extracellular proteolytic enzymes produced by human pathogenic vibrio species. *Front. Microbiol.* 4:339 10.3389/fmicb.2013.00339 PMC3831164 24302921

[B36] Moran N. A. McCutcheon J. P. Nakabachi A. (2008). Genomics and evolution of heritable bacterial symbionts. *Annu. Rev. Genet.* 42 165–190. 10.1146/annurev.genet.41.110306.130119 18983256

[B37] Moran N. A. Russell J. A. Koga R. Fukatsu T. (2005). Evolutionary relationships of three new species of *Enterobacteriaceae* living as symbionts of aphids and other insects. *Appl. Environ. Microbiol.* 71 3302–3310. 10.1128/AEM.71.6.3302-3310.2005 15933033 PMC1151865

[B38] Oliver K. M. Degnan P. H. Burke G. R. Moran N. A. (2010). Facultative symbionts in aphids and the horizontal transfer of ecologically important traits. *Annu. Rev. Entomol.* 55 247–266. 10.1146/annurev-ento-112408-085305 19728837

[B39] Oliver K. M. Smith A. H. Russell J. A. (2014). Defensive symbiosis in the real world – advancing ecological studies of heritable, protective bacteria in aphids and beyond. *Funct. Ecol.* 28 341–355. 10.1111/1365-2435.12133

[B40] Petersen L. M. Tisa L. S. (2013). Friend or foe? A review of the mechanisms that drive *Serratia* towards diverse lifestyles. *Can. J. Microbiol.* 59 627–640. 10.1139/cjm-2013-0343 24011346

[B41] Petersen L. M. Tisa L. S. (2014). Molecular characterization of protease activity in *Serratia* sp. strain SCBI and its importance in cytotoxicity and virulence. *J. Bacteriol.* 196 3923–3936. 10.1128/JB.01908-14 25182493 PMC4248818

[B42] Pfaffl M. W. (2001). A new mathematical model for relative quantification in real-time RT-PCR. *Nucleic Acids Res.* 29:e45 10.1093/nar/29.9.e45 PMC55695 11328886

[B43] Pons I. Renoz F. Noël C. Hance T. (2019). New insights into the nature of symbiotic associations in aphids: infection process, biological effects and transmission mode of cultivable *Serratia symbiotica* bacteria. *Appl. Environ. Microbiol.* 10.1128/AEM.02445-18 30850430 PMC6498149

[B44] Powell G. Tosh C. R. Hardie J. (2006). Host plant selection by aphids: behavioral, evolutionary, and applied perspectives. *Annu. Rev. Entomol.* 51 309–330. 10.1146/annurev.ento.51.110104.151107 16332214

[B45] Renoz F. Champagne A. Degand H. Morsomme P. Foray V. Hance T. (2017). Toward a better understanding of the mechanisms of symbiosis: a comprehensive proteome map a nascent insect symbiont. *PeerJ Preprints* 5:e3291. 10.7717/peerj.3291 28503376 PMC5426354

[B46] Renoz F. Noël C. Errachid A. Foray V. Hance T. (2015). Infection dynamic of symbiotic bacteria in the pea aphid *Acyrthosiphon pisum* gut and host immune response at the early steps in the infection process. *PLoS One* 10:e0122099. 10.1371/journal.pone.0122099 25811863 PMC4374939

[B47] Russell J. A. Latorre A. Sabater-Munoz B. Moya A. Moran N. A. (2003). Side-stepping secondary symbionts: widespread horizontal transfer across and beyond the Aphidoidea. *Mol. Ecol.* 12 1061–1075. 10.1046/j.1365-294X.2003.01780.x 12753224

[B48] Ryan C. A. (1990). Protease inhibitors in plants: genes for improving defenses against insects and pathogens. *Ann. Rev. Pathol.* 28 425–449. 10.1146/annurev.py.28.090190.002233

[B49] Sabri A. Leroy P. Haubruge E. Hance T. Frere I. Destain J. (2011). Isolation, pure culture and characterization of *Serratia symbiotica* sp. nov., the R-type of secondary endosymbiont of the black bean aphid *Aphis fabae*. *Int. J. Syst. Evol. Microbiol.* 61(Pt 9) 2081–2088. 10.1099/ijs.0.024133-0 20870890

[B50] Sabri A. Vandermoten S. Leroy P. D. Haubruge E. Hance T. Thonart P. (2013). Proteomic investigation of aphid honeydew reveals an unexpected diversity of proteins. *PLoS One* 8:e74656. 10.1371/journal.pone.0074656 24086359 PMC3783439

[B51] Shah J. Walling L. (2017). Editorial: advances in plant-hemipteran interactions. *Front. Plant Sci.* 8:1652. 10.3389/fpls.2017.01652 29033959 PMC5627026

[B52] Shevchenko A. Sunyaev S. Loboda A. Shevchenko A. Bork P. Ens W. (2001). Charting the proteomes of organisms with unsequenced genomes by MALDI-quadrupole time-of-flight mass spectrometry and BLAST homology searching. *Anal. Chem.* 73 1917–1926. 10.1021/ac0013709 11354471

[B53] Shevchenko A. Tomas H. Havlis J. Olsen J. V. Mann M. (2006). In-gel digestion for mass spectrometric characterization of proteins and proteomes. *Nat. Protoc.* 1 2856–2860. 10.1038/nprot.2006.468 17406544

[B54] Skaljac M. Kanakala S. Zanic K. Puizina J. Pleic I. L. Ghanim M. (2017). Diversity and phylogenetic analyses of bacterial symbionts in three whitefly species from Southeast Europe. *Insects* 8:E113. 10.3390/insects8040113 29053633 PMC5746796

[B55] Skaljac M. Kirfel P. Grotmann J. Vilcinskas A. (2018). Fitness costs of infection with *Serratia symbiotica* are associated with greater susceptibility to insecticides in the pea aphid *Acyrthosiphon pisum*. *Pest. Manag. Sci.* 74 1829–1836. 10.1002/ps.4881 29443436

[B56] Skaljac M. Zanic K. Ban S. G. Kontsedalov S. Ghanim M. (2010). Co-infection and localization of secondary symbionts in two whitefly species. *BMC Microbiol.* 10:142. 10.1186/1471-2180-10-142 20462452 PMC2877686

[B57] Stewart E. J. (2012). Growing unculturable bacteria. *J. Bacteriol.* 194 4151–4160. 10.1128/JB.00345-12 22661685 PMC3416243

[B58] Su Q. Oliver K. M. Xie W. Wu Q. Wang S. Zhang Y. (2015). The whitefly-associated facultative symbiont *Hamiltonella defensa* suppresses induced plant defences in tomato. *Funct. Ecol.* 29 1007–1018. 10.1111/1365-2435.12405

[B59] Sugio A. Dubreuil G. Giron D. Simon J. C. (2015). Plant-insect interactions under bacterial influence: ecological implications and underlying mechanisms. *J. Exp. Bot.* 66 467–478. 10.1093/jxb/eru435 25385767

[B60] Van Emden H. F. Harrington R. (2017). *Aphids as Crop Pests.* Wallingford: CABI 10.1079/9781780647098.0000

[B61] Wielkopolan B. Obrepalska-Steplowska A. (2016). Three-way interaction among plants, bacteria, and coleopteran insects. *Planta* 244 313–332. 10.1007/s00425-016-2543-1 27170360 PMC4938854

[B62] Will T. Schmidtberg H. Skaljac M. Vilcinskas A. (2017). Heat shock protein 83 plays pleiotropic roles in embryogenesis, longevity, and fecundity of the pea aphid *Acyrthosiphon pisum*. *Dev. Genes Evol.* 227 1–9. 10.1007/s00427-016-0564-1 27743033 PMC5203865

[B63] Wu J.-W. Chen X. L. (2011). Extracellular metalloproteases from bacteria. *Appl. Microbiol. Biotechnol.* 92:253. 10.1007/s00253-011-3532-8 21845384

[B64] Zhu-Salzman K. Zeng R. (2015). Insect response to plant defensive protease inhibitors. *Annu. Rev. Entomol.* 60 233–252. 10.1146/annurev-ento-010814-020816 25341101

