# Supplementary material for: Transmission of a Protease-Secreting Bacterial Symbiont Among Pea Aphids via Host Plants
Source: Front Physiol. 2019 Apr 17;10:438. doi: 10.3389/fphys.2019.00438 (PMC6479166; doi:10.3389/fphys.2019.00438)
Supplement: Supplementary file 1 [file Data_Sheet_1.docx]

Supplementary Material

**Transmission of a protease-secreting bacterial symbiont among pea aphids via host plants**

**Marisa Skaljac^1^, Heiko Vogel^2^, Natalie Wielsch^2^, Sanja Mihajlovic^1^ and Andreas Vilcinskas^1,3*^**

*** Correspondence:** Andreas Vilcinskas: Andreas.Vilcinskas@agrar.uni-giessen.de

**1 Supplementary Tables**

**Supplementary Table S1.** List of primers and probes.

| **Target (Organism/**  **Accession number*)** | **Purpose** | **E (%)** | **Product size (bp)** | **Primer sequence 5′→3′** | **Reference** |
| --- | --- | --- | --- | --- | --- |
| Serine endopeptidase (*DegP*)  (*S. symbiotica*/CDS55594.1) | cloning |  | 463 | Fwd ATCCAGACTGACGCCGCGAT  Rev TGGCGTCAAGGGTCATCGGT | This study |
| Serine endopeptidase (*DegQ*)  (*S. symbiotica*/CDS55928.1) | cloning |  | 408 | Fwd GGTCGGCCGCGATGAACAAT  Rev CTGCGCTTCACTTGGCCGAA |  |
|  | qPCR | E=92.9% R^2^=0.99 | 88 | Fwd GCGCCATGTTGCTTGGAATG  Rew GGCGAGTTGATCGGCATCAATA |  |
| Zn-dependent endopeptidase (*HtpX*)  (*S. symbiotica*/CDS58211.1) | cloning |  | 740 | Fwd CTGGCGGTGATGTTGGTTTT  Rev GCCATCAAGCTGCCTGTTTC |  |
|  | qPCR | E=92.5% R^2^=0.99 | 121 | Fwd CGCGCAACGAAACTGAGAATTG  Rev CCAGTGGCGAAGGCGTTAAT |  |
| Putative M48 family peptidase (*YfgC*)  (*S. symbiotica*/CDS57423.1) | cloning |  | 474 | Fwd GCCAGCGCGCCTTTAATCCA  Rev CGGATCGAAACCAGCGCGTT |  |
|  | qPCR | E=92.5% R^2^=0.99 | 123 | Fwd TCAATGCCTTCGCCTTCTTTGG  Rev GGCGTTGGGTAACGTGTGAAAT |  |
| Putative peptidase (*SohB*) (*S. symbiotica*/CDS58397.1) | cloning |  | 443 | Fwd ACCTTGCCTGTACGTGCTGGA  Rev TGAACTTCTCGCGGCCCTGT |  |
|  | qPCR | E=89.0% R^2^=0.99 | 101 | Fwd TAGCCTCCACGCAAGATGAAG  Rev CTTACGCAAGCGTTCCAACTG |  |
| Peptidase D (*PepD*)  (*S. symbiotica*/CDS55732.1) | cloning |  | 453 | Fwd GCGCATCGTTGGCACTGAGT  Rew AGTGTGGGCCGGTGATGGTT |  |
| Aminopeptidase A (*PepA*)  (*S. symbiotica*/CDS56273.1) | cloning |  | 406 | Fwd GATTGCCCAGAGAAACAGCG  Rev ACTGATCGAAGGTGTAAAGCGT |  |
|  | qPCR | E=90.5% R^2^=0.99 | 107 | Fwd ATCGCGGAACAACTCGACAAA  Rev GGCACATGGTGCAGCAGTAA |  |
| Aminopeptidase N (*PepN*) (*S. symbiotica*/CDS57483.1) | cloning |  | 412 | Fwd CATTGAAGCGGTGATTGGCC  Rev ATCTTCCATCGCCTGCACAA |  |
| Ribosomal protein L32 (*rpl32*)  (*A. pisum/*ACYPI000074) | qPCR (reference gene) | E=107.2% R^2^=0.99 | 131 | Fwd AGTATCGCCCAACAATTATCA  Rev CTTGAATCGTCTTCGGACT | Sapountzis *et al*., 2014 |
| Actin (*V. faba/*GQ339767.1) | qPCR (reference gene) | E=91.6% R^2^=0.99 | 108 | Fwd GAAGAGCTATGAGTTGCCTGAC  Rev CCTGCTGCTTCCATTCCTATC | This study |
| *Buchnera* (16*S* rRNA) | PCR |  | 430 | Fwd GAGCTTGCTCTCTTTGTCGGCAA  Rev CTTCTGCGGGTAACGTCACGAA | Tsuchida  *et al*., 2002 |
| *Serratia* (*dnaK*) | qPCR | E=95.0% R^2^=0.99 | 113 | Fwd TGGCGGGTGATGTGAAG  Rev CGGGATAGTGGTGTTTTTGG | Burke *et al*., 2009 |
| *Serratia* (16*S* rRNA) | PCR |  | 480 | Fwd AGAGTTTGATCMTGGCTCAG  Rev GCAATGTCTTATTAACACAT | Fukatsu &  Nikoh,  1998 |
|  | FISH |  |  | cy3-GACATCGTTTACAGCGTGGA | Luna-Ramirez *et al*., 2017 |

*Indicates GenBank number for sequences that were used to design primers for cloning; E-quantitative PCR efficiency;

R^2^-coefficient of determination

**Supplementary Table S2.** Detection of *S. symbiotica* in *A. pisum* and *V. faba* samples by fluorescence *in situ* hybridization.

| **Sample** | | **Presence(+)/absence(-) of *S. symbiotica*^§^** |
| --- | --- | --- |
| *V. faba* | Non-infested plant | - |
|  | Plant exposed to *Serratia*-positive aphids | + |
|  | Plant exposed to *Serratia*-free aphids | - |
| *Serratia*-positive aphids | Head section | + |
|  | Salivary glands | + |
|  | Gut | + |
| *Serratia*-free aphids | Head section | - |
|  | Salivary glands | - |
|  | Gut | - |

^§^Pool of insect specimens (head section, salivary glands, gut) (n=10) or *V. faba* tissues in at least three biological replicated were analysed by DNA extraction, PCR (*Serratia* (16*S* rRNA) and sequencing. The taxon was designated according to the bacterial genus of the BLAST hit with >96% sequence identity (nucleotide sequence accession numbers are provided in the Methods section).

**Supplementary Table S3.** Frequency of *S. symbiotica* found in *Serratia*-free aphids exposed to *V. faba* plants containing *S. symbiotica* (treatment) or *V. faba* free of *S. symbiotica* (control). The exposure time was 3 days.

| Replicates | **Treatment** | | **Control** | |
| --- | --- | --- | --- | --- |
|  | Number  of *Serratia*-free aphids exposed to *V. faba* containing *S. symbiotica* | % of aphids (*Serratia*-reinfected) infected with  *S. symbiotica* after 2 months from the infection event | Number of *Serratia*-free aphids exposed to *V. faba* free of *S. symbiotica* | % of aphids infected with  *S. symbiotica* |
| 5 | 30 | 100 | 30 | 0 |
| sum | 150 |  | 150 |  |

**Supplementary Table S4.** Complete list of proteolytic enzymes identified in *S. symbiotica* CWBI-2.3.

| **Protein identification^§^** | **GenBank accession number for top scoring protein/organism** | **Description** | **Corresponding band(s) in SDS-PAGE gel (Figure S7)** |
| --- | --- | --- | --- |
| Serine endopeptidase (*DegP*) | CDS55594.1/*S. symbiotica* | Membrane-associated | 13, 14 |
| Serine endopeptidase (*DegQ*) | CDS55928.1/*S. symbiotica* | Periplasmic | 14 |
| Putative IgA-specific serine endopeptidase | CDS57070.1/*S. symbiotica* | IgA-specific | 12 |
| Zn-dependent endopeptidase (*HtpX*) | CDS58211.1*/S. symbiotica* | Membrane-associated; self-cleaved (heat shock protein HtpX) | 7, 8 |
| Putative M48 family peptidase (*YfgC*) | CDS57423.1/*S. symbiotica* |  | 14 |
| Putative peptidase (*SohB*) | CDS58397.1/*S. symbiotica* | Inner membrane peptidase | 11 |
| Peptidase D (*PepD*) | CDS55732.1/*S. symbiotica* | Aminoacyl-histidine dipeptidase | 14 |
| Aminopeptidase A  (*PepA*) | CDS56273.1/*S. symbiotica* | Cysteinylglycinase | 14, 15 |
| Aminopeptidase N (*PepN*) | CDS57483.1/*S. symbiotica* |  | 18 |
| Dipeptidase | AGE16693.1/*S. marcescens* | Aminoacyl-histidine dipeptidase | 14 |
| Dipeptidase | EMF07010.1/*S. marcescens* | Aminoacyl-histidine dipeptidase | 14 |
| Putative protease | CDS58954.1/*S. symbiotica* | Membrane anchored | 10, 11 |
| Synthetase protease | CDS57462.1/*S. symbiotica* | Glutamyl-tRNA synthetase | 14 |
| Chaperone ClpB protease | CDS57673.1/*S. symbiotica* | ATP-binding protease component | 18 |
| Chaperone ClpB protease | CDS59016.1/*S. symbiotica* | Disaggregation ATP-dependent | 16, 17 |

**^§^**Some periplasmic and membrane-associated enzymes also appear on this list because the corresponding proteins are occasionally found in the extracellular environment due to processes such as transcytosis, or natural phenomena such as cell lysis.

**Supplementary Table S5.** Summary of qPCR/qRT-PCR data obtained in this study as shown in Figures 2 and 3.

| **Tracking parameters** | **Gene**  **(*S. symbiotica)*** | **Abundance ratio^§^** | | | | | | **Significance** |
| --- | --- | --- | --- | --- | --- | --- | --- | --- |
|  |  | ***Serratia*-positive** | | | ***Serratia*-free** | | |  |
| Abundance of *S. symbiotica* in the two aphid lines | *dnaK* | 328652.35±  30137.77 | | | 1.01±  0.12 | | | p < 0.001 |
| Retention time of *S. symbiotica* in *V. faba* leaves infested by the two aphid lines | *dnaK* | **2 d** | **5 d** | **10 d** | **2 d** | **5 d** | **10 d** | (2d) p < 0.01  (5 d) p < 0.05  (10 d) p< 0.01 |
|  |  | 365.63  ±141.09 | 198.17  ±103.13 | 5.31  ±2.12 | 1.23  ±0.41 | 1.19  ±0.47 | 1.23  ±0.58 |  |
| Expression of *S. symbiotica* protease genes in the two aphid lines | Serine endopeptidase (*DegQ*) | 6756.47  ±4219.45 | | | 1.06±  0.36 | | | p < 0.05 |
|  | Zn-dependent endopeptidase (*HtpX*) | 302.56  ±199.52 | | | 1.02  ±0.19 | | | p < 0.01 |
|  | Putative M48 family peptidase (*YfgC*) | 655.56  ±410.99 | | | 1.01  ±0.10 | | | p < 0.05 |
|  | Putative peptidase (*SohB*) | 2233.08  ±1376.68 | | | 1.22  ±0.72 | | | p < 0.05 |
|  | Aminopeptidase A  (*PepA*) | 709.05  ±482.92 | | | 1.07  ±0.39 | | | p < 0.05 |

^§^Mean±SE; d-day; the two aphid lines refer to *Serratia*-free and *Serratia*-positive lines.

**2 Supplementary Figures**

**
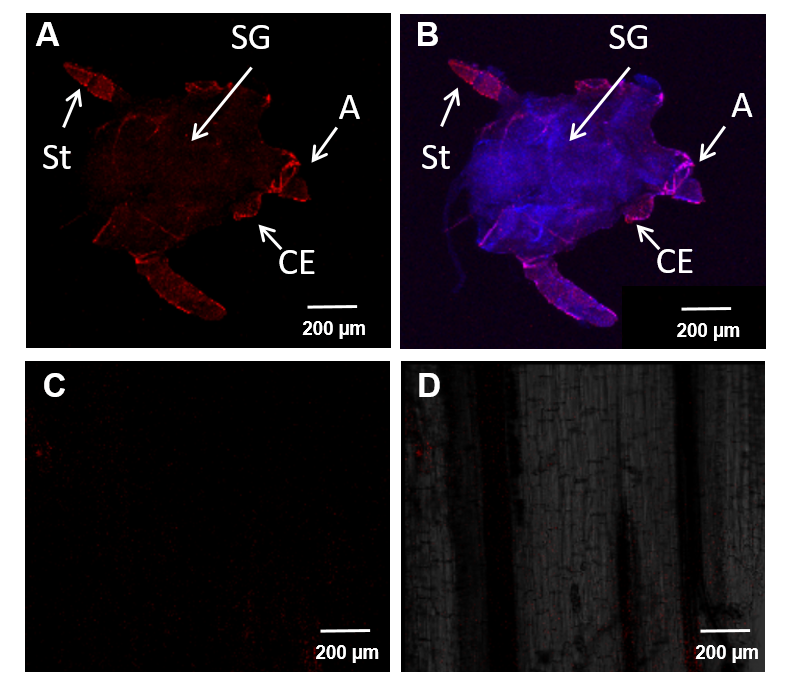
**

**Supplementary Figure S1.** Fluorescence *in situ* hybridization, showing negative controls in *Serratia*-free *A. pisum* head and *V. faba* tissues that were exposed to non-infected aphids. *S. symbiotica* was not localized in the head (mouthparts, salivary glands) of a 10-day-old adult (A-B) and *V. faba* longitudinal stem sections under dark field (A-C) and bright field (D) imaging. Nuclei of aphids were counterstained with DAPI (dark blue). Abbreviations: A = antennae; SG = salivary gland; CE = compound eye.


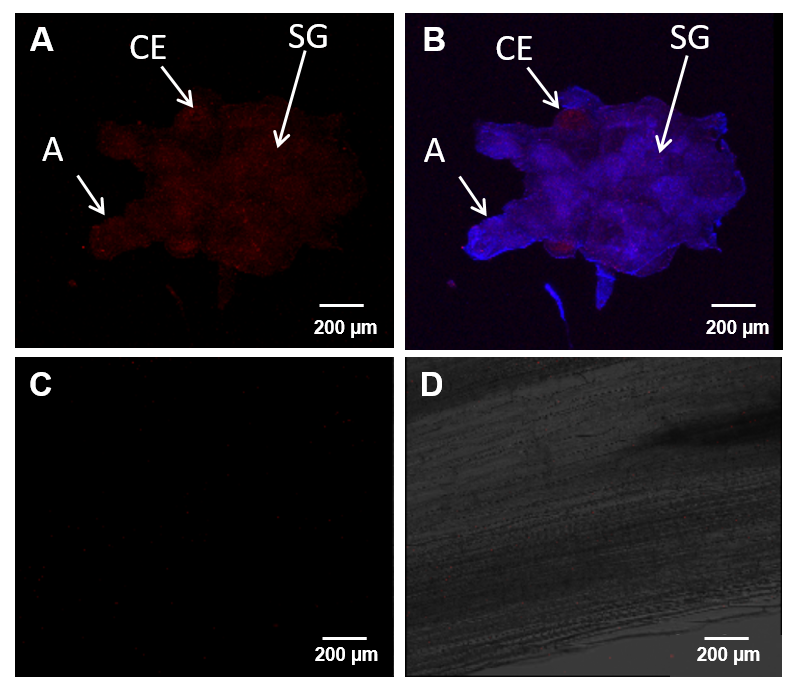


**Supplementary Figure S2.** Fluorescence *in situ* hybridization, showing negative controls (no-probe staining) in *Serratia*-positive *A. pisum* head (mouthparts, salivary glands) and *V. faba* tissues that were exposed to infected aphids. Dark field (A-C) and bright field (D) imaging. Nuclei of aphids were counterstained with DAPI (dark blue). Abbreviations: A = antennae; SG = salivary gland; CE = compound eye.

**
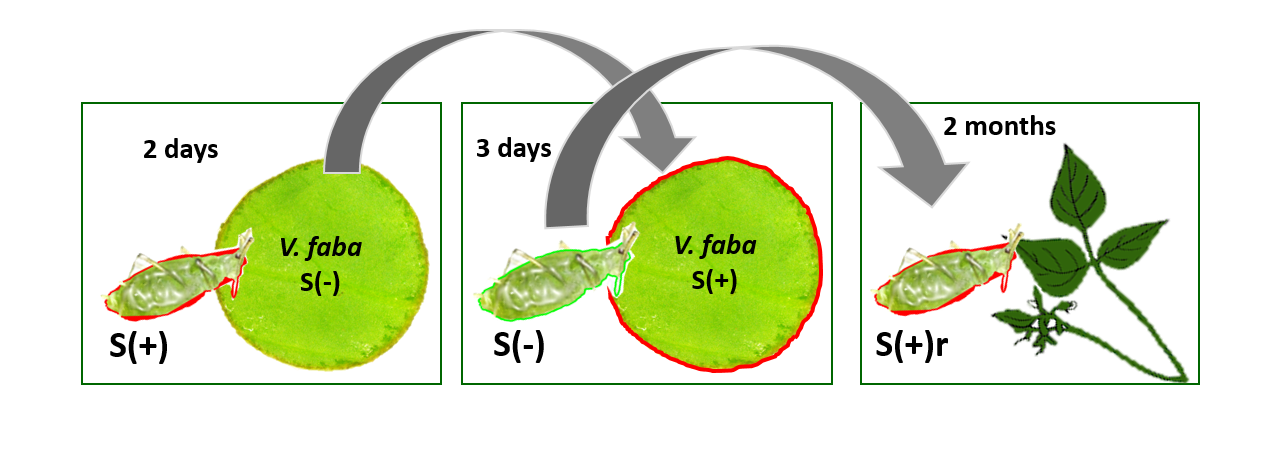
**

**Supplementary Figure S3.** Scheme representing the horizontal transmission of *S. symbiotica* via plants. For a detailed description of the methodology, see Section 2.1.3 “Horizontal transmission of *S. symbiotica* between *A. pisum* individuals via host plants”. S(+) = *Serratia*-positive aphids or *V. faba*; S(-) = *Serratia*-free aphids or *V. faba*; S(+)r = *Serratia*-reinfected aphids. *Serratia*-positive aphids were inoculated onto *V. faba* (leaf discs) for 2 days then removed, and *Serratia*-free aphids were allowed to feed on the infected area of the leaf discs for the next 3 days and then left for another 2 months for infection to spread among the aphid population. All aphids (*Serratia*-reinfected) in the population were found to be infected with the symbiont.

**
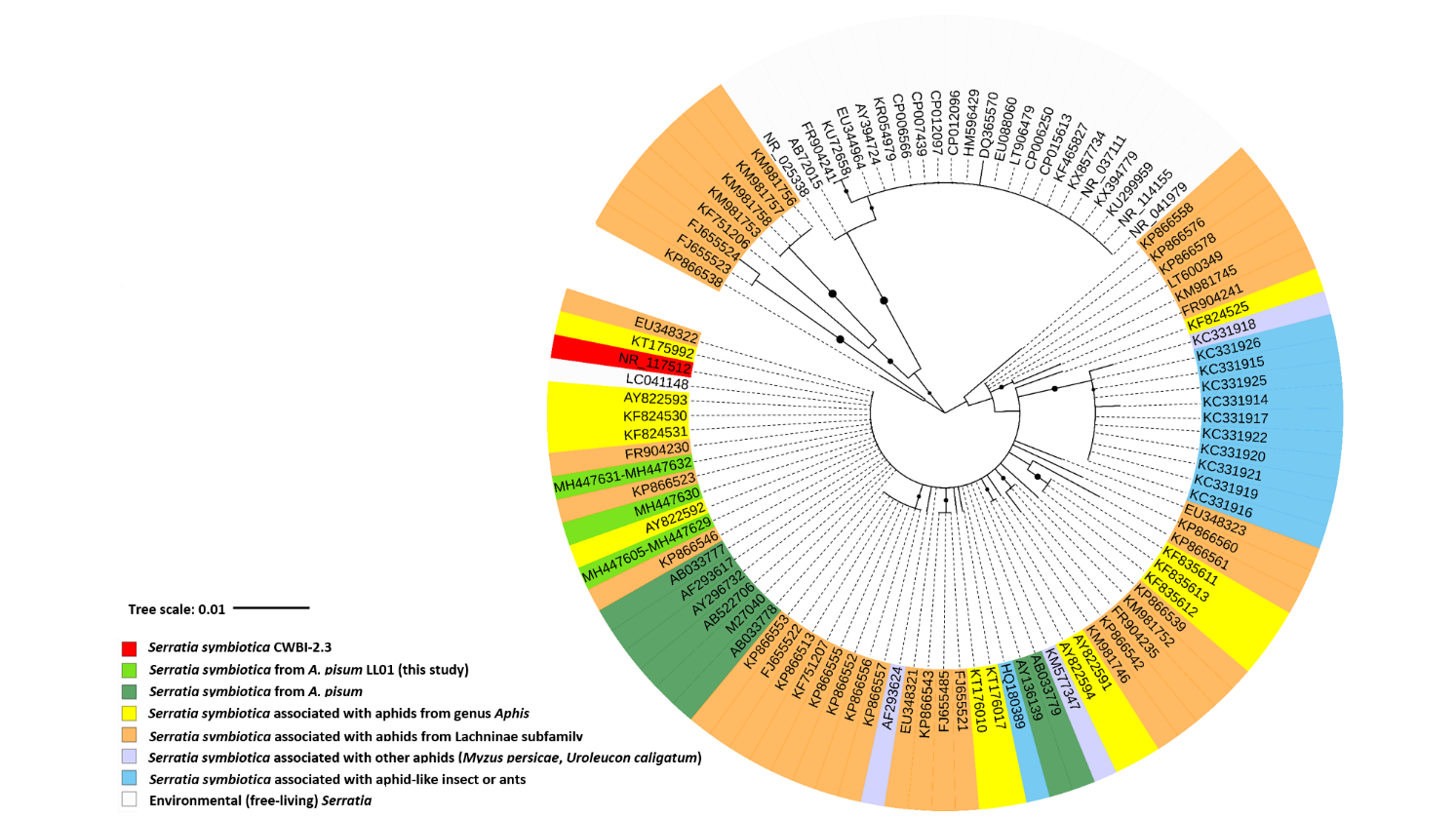
**

**Supplementary Figure S4.** Phylogenetic relationships of the *S. symbiotica* strains identified in this study. The phylogenetic tree was constructed using the maximum-likelihood method based on 16*S* rDNA sequences from GenBank and this study. Bootstrapping of 1000 resamples was used to infer confidence levels. Black circles represent the nodes with a support bootstrap value of ≥ 50%. GenBank accession numbers of 16*S* rDNA sequences are shown in the tree, whereas the colour code represents aphid taxa. Bar = 0.01 substitutions per nucleotide position.

**
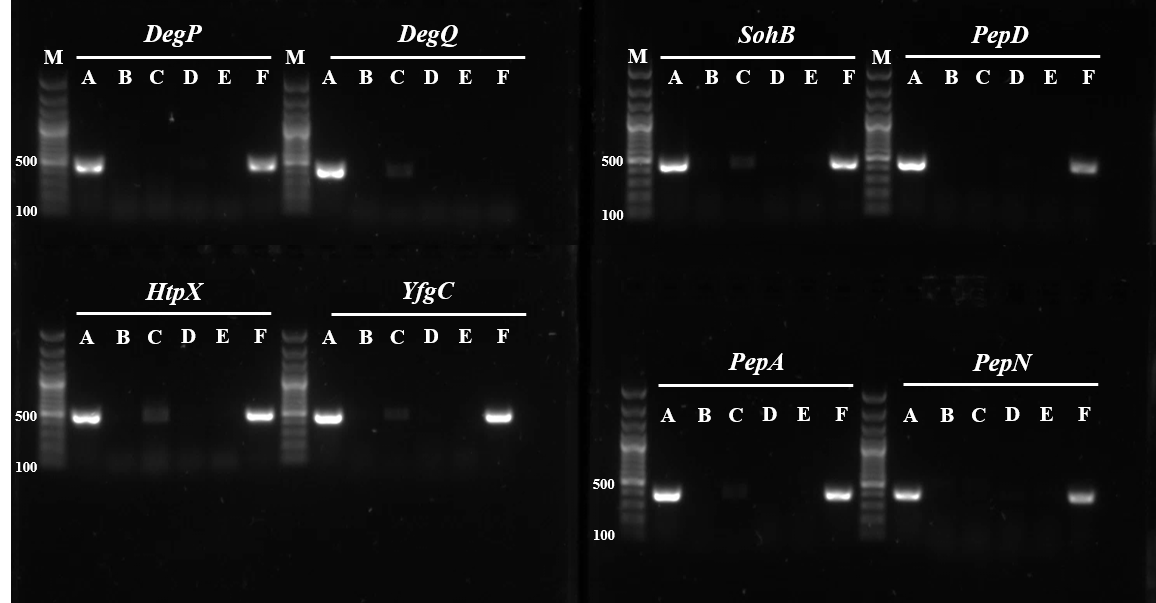
**

**Supplementary Figure S5.** Amplicons of transcripts representing genes encoding proteolytic enzymes from the two *S. symbiotica* strains in this study (for additional details, see Table 1). *DegP*, serine endopeptidase; *DegQ*, serine endopeptidase; *HtpX*, Zn-dependent endopeptidase; *YfgC*, putative M48 family peptidase; *SohB*, putative peptidase; *PepD*, peptidase D; *PepA*, aminopeptidase A; *PepN* aminopeptidase N. Samples shown in the figure: A, *Serratia-*positive aphids; B, *Serratia-*free aphids; C, *V. faba* infested with *Serratia-*positive aphids; D, *V. faba* infested with *Serratia-*free aphids; E, non-infested *V. faba*; F, *S. symbiotica* CWBI-2.3. Amplicons of *S. symbiotica* genes encoding proteolytic enzymes (Table 1) from *V. faba* infested with *Serratia-*positive aphids (C) were re‑amplified and cloned, followed by sequencing together with amplicons from *Serratia-*positive aphids (A) and *S. symbiotica* CWBI-2.3 (F).


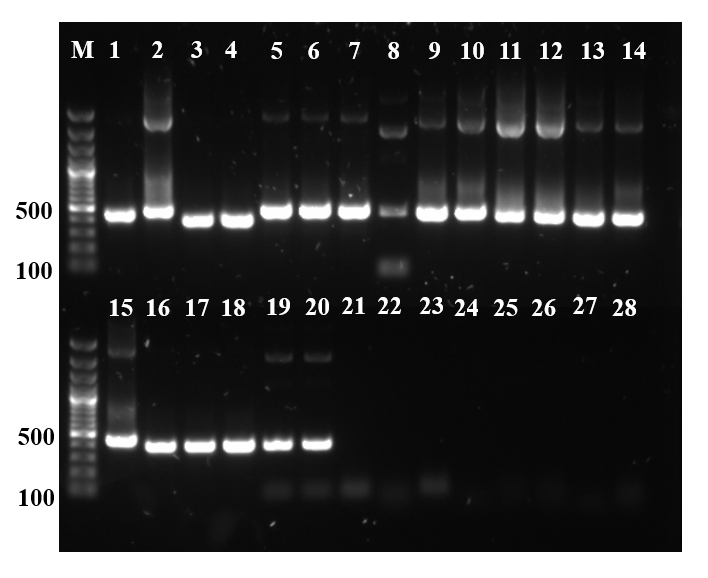


**Supplementary Figure S6.** Vector pGEM T-Easy containing protease gene amplicons investigated in this study. M, DNA marker (size in base pairs); lane 1, *Deg P* from *Serratia*-positive aphids; lane 2, *Deg P* from *Serratia* *symbiotica* CWBI-2.3; lane 3, *DegQ* from *Serratia*-positive aphids; lane 4, *DegQ* from *V. faba* carrying *S. symbiotica*; lane 5, *HtpX* from *Serratia*-positive aphids; lane 6, *HtpX* from *V. faba* carrying *S. symbiotica*; lane 7, *HtpX* from *S. symbiotica* CWBI-2.3; lane 8, *YfgC* from *Serratia*-positive aphids; lane 9, *YfgC* from *V. faba* carrying *S. symbiotica*; lane 10, *YfgC* from *S. symbiotica* CWBI-2.3; lane 11, *SohB* from *Serratia*-positive aphids; lane 12, *SohB* from *V. faba* carrying *S. symbiotica*; lane 13, *SohB* from *S. symbiotica* CWBI-2.3; lane 14, *PepD* from *Serratia*-positive aphids; lane 15, *PepD* from *S. symbiotica* CWBI-2.3; lane 16, *PepA* from *Serratia*-positive aphids; lane 17, *PepA* from *V. faba* carrying *S. symbiotica*; lane 18, *PepA* from *S. symbiotica* CWBI-2.3; lane 19, *PepN* from *Serratia*-positive aphids; lane 20, *PepN* from *S. symbiotica* CWBI-2.3; lanes 21-28, negative controls (distilled water) used in PCRs for each protease-related primer pair in Table S1. Details of primers and amplicons are provided in Table S1. GenBank accession numbers for each amplicon/sequence are listed in Table 1.

**
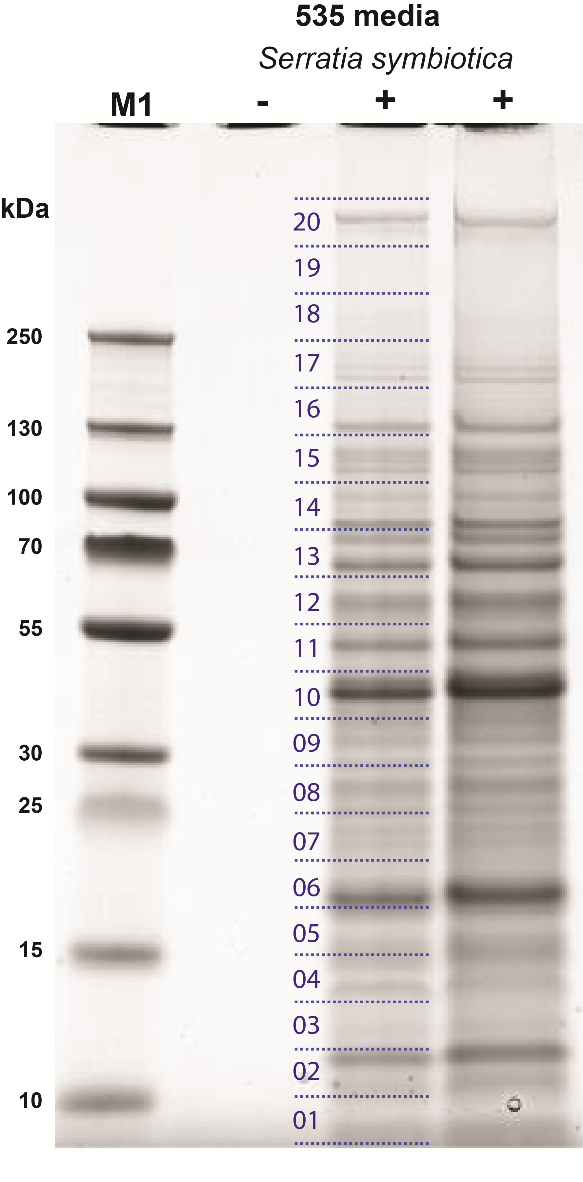
**

**Supplementary Figure S7.** SDS-PAGE analysis of extracellular proteins in the *S. symbiotica* CWBI-2.3 culture supernatant. Proteins obtained from the supernatant of *S. symbiotica* CWBI-2.3 cultured in 535 medium, were separated by SDS-PAGE and stained with Coomassie Brilliant Blue R250. M = marker; (-) = medium-only control; (+) = replicate of the bacterial supernatant.

**References**

Burke, G.; Fiehn, O.; Moran, N. Effects of facultative symbionts and heat stress on the metabolome of pea aphids. *The ISME journal*. **2010**, 4(2), 242.

Tsuchida, T.; Koga, R.; Shibao, H.; Matsumoto, T.; Fukatsu, T. Diversity and geographic distribution

of secondary endosymbiotic bacteria in natural populations of the pea aphid, *Acyrthosiphon pisum*. *Mol. Ecol.* **2002**, *11*, 2123–2135.

Fukatsu, T.; Nikoh, N. Two intracellular symbiotic bacteria from the mulberry psyllid *Anomoneura*

*mori* (Insecta, Homoptera). *Appl. Environ. Microbiol.* **1998**, *64*, 3599–3606.

Luna-Ramirez, K.; Skaljac, M.; Grotmann, J.; Kirfel, P.; Vilcinskas, A. Orally Delivered Scorpion Antimicrobial Peptides Exhibit Activity against Pea Aphid (*Acyrthosiphon pisum*) and Its Bacterial Symbionts. *Toxins*. **2017**, 9(9).
